# Two Birds With One Stone: The Decisive Role of Cardiac MRI in Identifying Both Hypertrophic Cardiomyopathy and Pericarditis Simultaneously in a Patient with Chest Pain

**DOI:** 10.7759/cureus.10843

**Published:** 2020-10-07

**Authors:** Islam M Shatla, Shahbaz Malik, Ali A Malhi, Ata Ur Rahim Bajwa

**Affiliations:** 1 Internal Medicine, University of Missouri Kansas City (UMKC), Kansas, USA; 2 Internal Medicine, Kansas City University of Medicine and Bioscience (KCUMB), Kansas, USA; 3 Emergency Medicine, Wayne State University Detroit Medical Center, Detroit, USA; 4 Cardiology, Penn State University College of Medicine, Milton S. Hershey Medical Center, Hershey, USA

**Keywords:** hypertrophic obstructive cardiomyopathy, cardiac magnetic resonance

## Abstract

A 35-year-old Hispanic male presented at an outside facility with chest pain a few days after a long road trip. The initial electrocardiogram (EKG) showed sinus tachycardia with no other abnormality. His D-dimer was positive but a subsequent computed tomography angiography (CTA) of the chest was negative for pulmonary embolism. An echocardiogram showed trace pericardial effusion with a normal ejection fraction (EF) of 70% and severe asymmetric septal hypertrophy. Satisfactory Doppler signals to assess the gradient across the left ventricle outflow tract (LVOT) could not be obtained on echocardiogram. The patient was diagnosed with acute pericarditis, which was treated medically with an improvement of his symptoms. Later, he presented to our facility for an outpatient cardiac magnetic resonance (CMR) with and without contrast, which showed severe asymmetric septal hypertrophy measuring 29 mm with substantial patchy myocardial delayed enhancement and systolic anterior motion of the mitral leaflet with flow dephasing of LVOT. These findings were diagnostic of hypertrophic obstructive cardiomyopathy. CMR also showed signs consistent with pericarditis. A Holter monitor was unremarkable for arrhythmia. A stress echocardiogram did not demonstrate any drop in blood pressure during exercise. His interventricular septum measured 29 mm on cardiac magnetic resonance imaging (MRI), which was very close to the 30 mm cut-off for an implantable cardioverter-defibrillator (ICD). In addition, he had a marked delayed enhancement in the hypertrophied septum due to gadolinium uptake, which is also considered a high-risk feature for sudden cardiac death. After discussions between the patient, cardiologist, cardiac imaging specialist, and electrophysiologist, a subcutaneous ICD was pursued, which was successfully implanted. He was started on medical treatment. He was followed closely in the clinic and has remained asymptomatic for the past two years.

## Introduction

Echocardiography remains the most commonly used imaging modality for many cardiac conditions, however, cardiac magnetic resonance imaging (MRI) is an emerging modality with complementary and additive data to echocardiography findings [1]. Hypertrophic cardiomyopathy (HCM) can be clinically silent or symptomatic [2]. Even if asymptomatic, it can still exhibit the high-risk features associated with sudden cardiac death and, therefore, timely diagnosis is critical. Early diagnosis and proper intervention can improve survival in these patients [3].

## Case presentation

**Figure 1:**
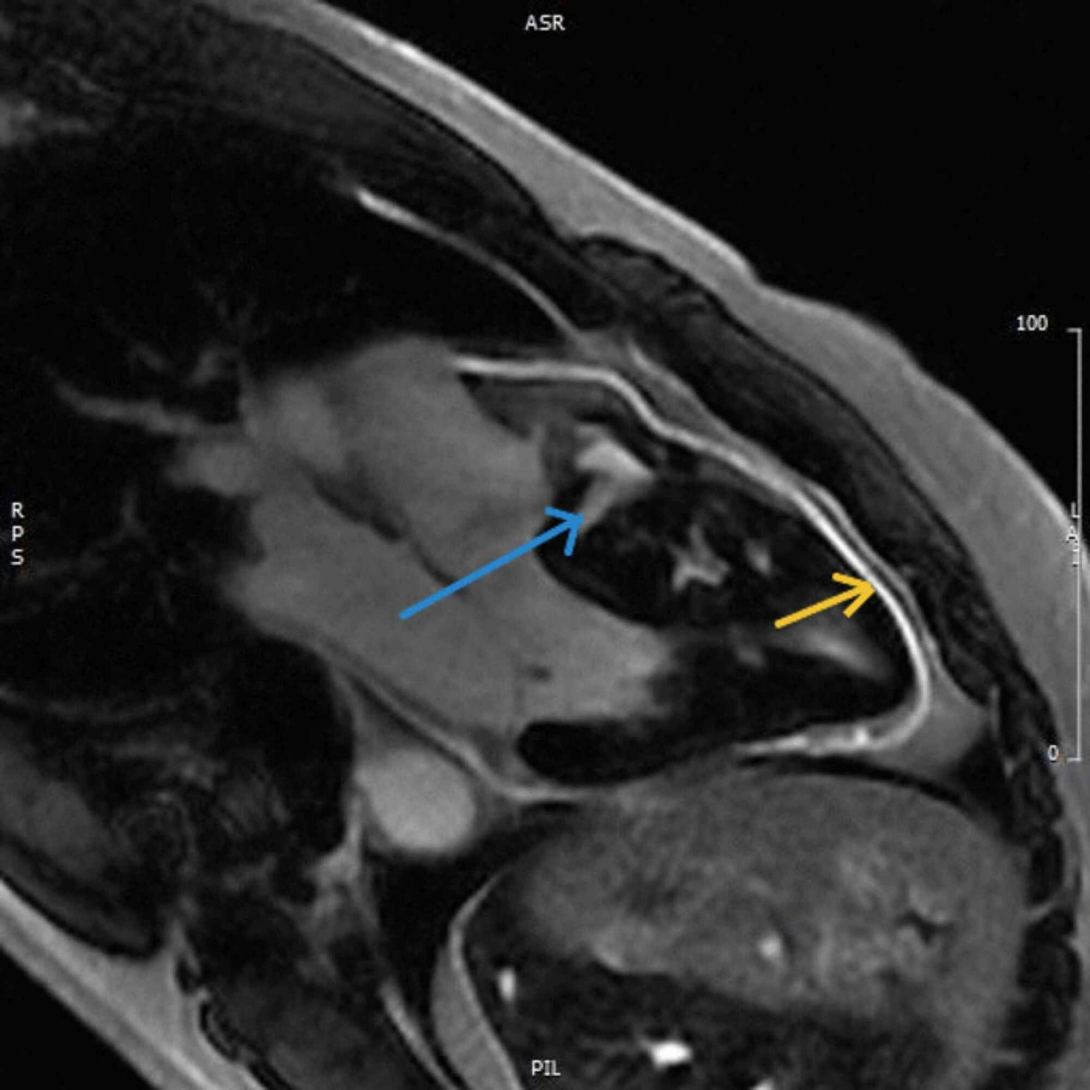
The blue arrow points to the hypertrophied septum while the yellow arrow points to the pericardium, both demonstrating delayed enhancement after gadolinium uptake

**Figure 2:**
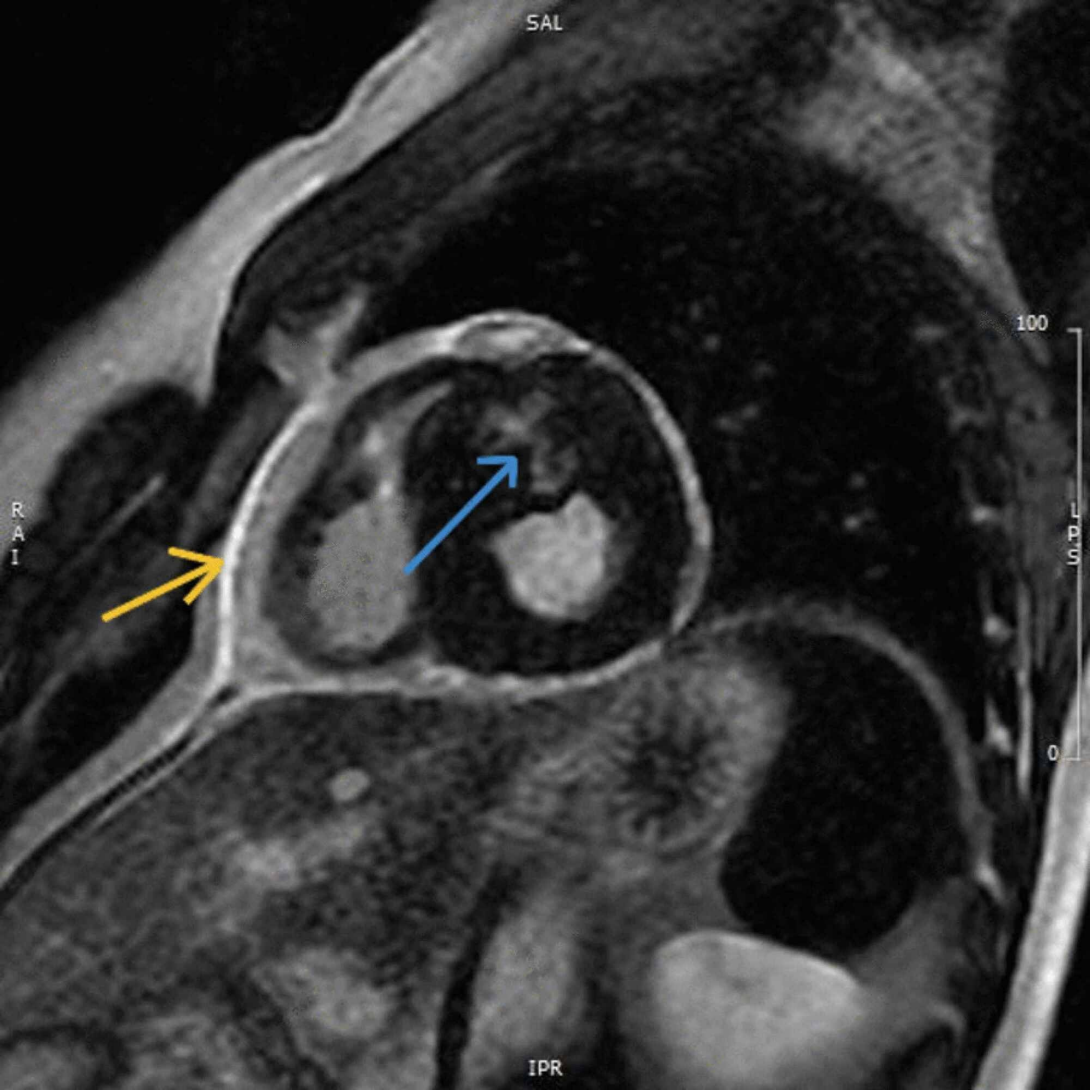
The blue arrow points to the hypertrophied septum while the yellow arrow points to the pericardium, both demonstrating delayed enhancement after gadolinium uptake These findings were diagnostic of severe hypertrophic cardiomyopathy in the setting of pericarditis.

A 35-year-old Hispanic male with a past medical history of ulcerative colitis (UC) presented at an outside facility with left-sided pleuritic chest pain a few days after a long road trip. The initial electrocardiogram (EKG) showed sinus tachycardia with no other significant findings. His D-dimer was positive but a subsequent computed tomography angiography (CTA) of the chest was negative for pulmonary embolism. The EKG showed trace pericardial effusion with a normal ejection fraction (EF) of 70% and severe asymmetric septal hypertrophy. Satisfactory Doppler signals to assess the gradient across the left ventricle outflow tract (LVOT) could not be obtained on EKG. The patient was diagnosed with acute pericarditis, given the overall findings, as well as elevated inflammatory markers of erythrocyte sedimentation rate (ESR) and C-reactive protein (CRP). He was discharged with analgesic medications and his chest pain resolved within a few days. Later, he presented to our facility for an outpatient cardiac magnetic resonance (CMR) with and without contrast. This study demonstrated severe asymmetric septal hypertrophy measuring 29 mm, with substantial patchy myocardial delayed enhancement (blue arrows in Figure 1 and Figure 2) and systolic anterior motion of the mitral leaflet with flow dephasing of LVOT. These findings were diagnostic of hypertrophic obstructive cardiomyopathy. Cardiac MRI was also remarkable for increased pericardial thickness with marked pericardial delayed enhancement consistent with pericarditis (yellow arrows in Figure 1 and Figure 2).

A Holter monitor demonstrated occasional PVCs without any non-sustained or sustained ventricular tachycardia (VT). A stress echocardiogram shows the left ventricular outflow tract gradient at 11 mmHg at rest and an increase to a maximal peak gradient of 42 mmHg after Valsalva and 34 mmHg after exercise but did not demonstrate any drop in blood pressure during exercise.

Decision‐Making

Other than pleuritic chest pain, the patient did not have any worrisome symptoms. He did have an episode of syncope a few months ago, which was reportedly orthostatic and happened in the setting of severe anemia. His interventricular septum measured 29 mm on cardiac MRI, which was very close to the 30 mm cut-off for an implantable cardioverter-defibrillator (ICD). In addition, he had marked delayed enhancement in the hypertrophied septum due to gadolinium uptake, which is also considered a high-risk feature for sudden cardiac death.

After discussions between the patient, cardiologist, cardiac imaging specialist, and electrophysiologist, a subcutaneous ICD was pursued, which was successfully implanted. He was also started on metoprolol extended-release 50 mg daily, given evidence of resting obstruction on CMR. Genetic screening was also performed, which showed a gene mutation of uncertain significance. Regardless, he was advised for the screening of family members. He was further advised to remain well-hydrated and avoid heavy exertional activities. The patient has been closely followed and, over the course of two years, has remained symptom-free. Follow-up transthoracic echocardiogram (TTE) showed hyperdynamic left ventricular systolic function, with EF=75%, severe asymmetric septal hypertrophy, with a mid-left ventricle (LV) resting peak gradient of 15 mmHg and 125 mmHg on Valsalva. Now he exercises several days per week; he notes occasional palpitation without syncope or ICD discharges.

## Discussion

Although echocardiography provides valuable data about different heart structures, including the pericardium, myocardium, and endocardium [4-5], it has some limitations such as poor acoustic window due to underlying chronic obstructive pulmonary disease (COPD) or body habitus, limited tissue characterization, and high operator dependence; hence the need for new imaging modalities [6].

CMR imaging is considered a relatively new non-invasive imaging modality that allows the assessment of both cardiac structures and function. CMR is useful in diagnosing different cardiovascular diseases, including ischemic heart disease, pericardial, myocardium, congenital heart disease, as well as valvular heart disease. Our discussion is focused on the role of CMR in the assessment of pericardial diseases as well as HCM.

CMR enables a more detailed evaluation of pericardial diseases such as pericarditis, non-calcified constrictive pericarditis, and metastatic tumors to pericardium [7-9]. The inflammatory process of the pericardium is usually seen as pericardial enhancement after intravenous gadolinium administration [10]. Late gadolinium enhancement detects pericardial inflammation with excellent sensitivity reaching (94%-100%) [11-12]; in addition, its extent can guide the initiation of anti-inflammatory therapy. Concomitant myocarditis can be also diagnosed with CMR.

A clinical diagnosis of HCM is confirmed when increased LV wall thickness ≥15 mm is detected by imaging [13]. Both CMR and echocardiography are valuable imaging modalities in the assessment of HCM patients. Echocardiography is a commonly used imaging modality to diagnose HCM, with findings such as LVH, increased LVOT gradient, and systolic anterior motion of the mitral valve leaflets.

CMR is considered a reliable imaging modality for the diagnosis and management of HCM, as it provides accurate assessment for left ventricular thickness, degree of interstitial fibrosis, and late gadolinium enhancement extent, which is an independent predictor of sudden cardiac death.

It was reported that there is a twofold increase in sudden death risk in HCM patients who have extensive enhancement ≥15 percent of LV mass even if they do not have other conventional sudden death markers [14]. Also, interstitial fibrosis is common among these patients with a reported range between 33%-86% across studies [15-17]. Our patient was diagnosed with pericarditis and HCM with the aid of CMR. He also had an ICD placed based on the high-risk features of his septal thickness and late gadolinium enhancement detected with CMR.

## Conclusions

This case demonstrates that HCM may not manifest any symptoms but may still have high-risk features for sudden cardiac death. Our patient presented with acute pericarditis and was incidentally diagnosed with severe HCM. CMR is a unique imaging tool not only for HCM diagnosis but also for management.
